# Optimisation of Recombinant Production of Active Human Cardiac SERCA2a ATPase

**DOI:** 10.1371/journal.pone.0071842

**Published:** 2013-08-12

**Authors:** Ana V. Antaloae, Cédric Montigny, Marc le Maire, Kimberly A. Watson, Thomas L.-M. Sørensen

**Affiliations:** 1 School of Biological Sciences, Whiteknights Campus, University of Reading, Reading, Berkshire, United Kingdom; 2 CEA, iBiTec-S, CNRS, UMR 8221, Universite Paris-Sud, Saclay, France; 3 Diamond Light Source Ltd., Diamond House, Harwell Science and Innovation Campus, Didcot, Oxfordshire, United Kingdom; Griffith University, Australia

## Abstract

Methods for recombinant production of eukaryotic membrane proteins, yielding sufficient quantity and quality of protein for structural biology, remain a challenge. We describe here, expression and purification optimisation of the *human* SERCA2a cardiac isoform of Ca^2+^ translocating ATPase, using *Saccharomyces cerevisiae* as the heterologous expression system of choice. Two different expression vectors were utilised, allowing expression of C-terminal fusion proteins with a biotinylation domain or a GFP- His_8_ tag. Solubilised membrane fractions containing the protein of interest were purified onto Streptavidin-Sepharose, Ni-NTA or Talon resin, depending on the fusion tag present. Biotinylated protein was detected using specific antibody directed against SERCA2 and, advantageously, GFP-His_8_ fusion protein was easily traced during the purification steps using in-gel fluorescence. Importantly, talon resin affinity purification proved more specific than Ni-NTA resin for the GFP-His_8_ tagged protein, providing better separation of oligomers present, during size exclusion chromatography. The optimised method for expression and purification of *human* cardiac SERCA2a reported herein, yields purified protein (> 90%) that displays a calcium-dependent thapsigargin-sensitive activity and is suitable for further biophysical, structural and physiological studies. This work provides support for the use of *Saccharomyces cerevisiae* as a suitable expression system for recombinant production of multi-domain eukaryotic membrane proteins.

## Introduction

Cellular calcium homeostasis is regulated by a large number of proteins, with the Sarco/Endoplasmic Reticulum Ca^2+^-ATPase (SERCA) pumps as key players in this process. SERCA pumps are integral membrane proteins responsible for Ca^2+^ uptake from the cytosol into the sarcoplasmic/endoplasmic reticulum (SR/ER), using the energy derived from ATP hydrolysis to fuel the ion translocation. This process is vital for preserving low intracellular calcium levels in a resting cell, a prerequisite for the use of calcium as a secondary messenger to control essential cellular processes such as muscle relaxation/contraction, cell signalling and apoptosis. *Human* SERCA (*h*SERCA) pumps belong to the P-type ATPase superfamily and are encoded by three different genes (ATP2A1, ATP2A2 and ATP2A3), each with its splice variants, giving rise to SERCA1a-b, SERCA2a-c and SERCA3a-f isoforms. All SERCAs have a total molecular weight of about 110 kDa and share a similar general structural organization, possessing an integral transmembrane domain and a large cytoplasmic domain. The cytoplasmic domain contains three distinct subdomains; the nucleotide binding domain (N), the phosphorylation domain (P) and the actuator domain (A). These subdomains are jointly responsible for ATP binding and hydrolysis, and serve as the motor driving ion translocation through long-range intra-molecular interactions with the integral membrane domain. The integral membrane domain consists of ten, or for SERCA2b eleven, transmembrane (TM) helices and is responsible for calcium binding and translocation [1,2].

All SERCA isoforms are homologous; the main amino-acid sequence differences are located at the C-terminal ends. Functionally, SERCA isoforms have different affinities for Ca^2+^ and different enzymatic turn-over rates [3–5].

Mammalian SERCA1a isoform is present mainly in adult fast-twitch muscle and mammalian SERCA1b is found in foetal muscle tissue, whereas mammalian SERCA3 isoforms (a-f) have been found to be expressed in various tissues: heart, skin, platelets and pancreas [6–10]. Rabbit SERCA1a (*r*SERCA1a) has been extensively studied since the 1960s [11–15], its abundance in muscle tissue has made it the source of choice for functional and structural analyses and it has been used as a benchmark for recombinant eukaryotic protein expression and purification from *S. cerevisiae* [16–18].

Three *human* SERCA2 isoforms have been identified: *h*SERCA2a, found mainly in the heart and slow-twitch muscle [19,20]; *h*SERCA2b, ubiquitously expressed and is present in neurons and epidermis [21]; and *h*SERCA2c is found in pancreatic, hepatic and mesenchymal cells [20,22,23]. Genetic engineering experiments revealed that SERCA2b has an extra transmembrane domain compared to SERCA1a and a longer C-terminal luminal tail, providing the highest affinity for Ca^2+^ and the lowest enzymatic turn-over [24]. Physiologically, SERCA2a is the main Ca^2+^ translocating isoform in the heart, its activity being controlled by other important proteins such as phospholamban and sarcolipin [25].

Numerous studies have shown a correlation between SERCA2a down-regulation and heart failure [26–28], with specific mutations in *h*SERCA2a and *h*SERCA2b leading to skin diseases without affecting heart activity [29,30]. Recently, a biotechnology company, Celladon (www.celladon.net) has over-expressed SERCA2a protein by transgenic technologies and is currently performing phase II clinical trials for humans suffering from heart failure, with positive results.

To study *h*SERCA2a in greater detail and to understand the complex nature of the physiological regulation in the heart, a method is required for expression and purification of *h*SERCA2a, in sufficient quantities and purity, suitable for in depth functional and structural characterisation, similar to methods developed for other complex membrane proteins [18,31,32].

We present herein an optimised route for recombinant production of *h*SERCA2a in *S. cerevisiae* purified by two successive purification steps to obtain enzymatically active protein suitable for crystallisation trials and biophysical characterisation. The functional analysis of the purified protein demonstrates Ca^2+^-dependent ATP consumption and thapsigargin inhibition, confirming that the recombinant purified protein is correctly folded and active.

## Materials and Methods

### Materials

All reagents used were obtained from Sigma-Aldrich, UK unless stated otherwise. Restriction enzymes and polymerase were purchased from New England Biolabs and Promega. Talon resin was from Clontech and Ni-NTA super-flow resin was from Qiagen. Streptavidin Sepharose High Performance resin was from GE Healthcare and Thrombin from Calbiochem. Complete EDTA free protease inhibitor was from Roche. 1,2 –dioleoyl-sn-Glycero-3-Phosphocholine (DOPC) was from Avanti Polar Lipids. Dodecyl-β-D-maltoside (DDM) was from Glycon Biochemicals. Octaethylene glycol monododecyl ether (C_12_E_8_) was from Nikko Chemicals. 4-12% Tris-Glycine SDS-PAGE precast gels were from Invitrogen. Bio-Rad_DC_ Protein assay was from Bio-Rad Laboratories. cDNA for *human* SERCA2a (NM_001681) was a gift from Anne-Marie Lompré (Inserm UMRS956/Université Pierre et Marie Curie). Antibodies were purchased from Santa Cruz Biotechnologies.

### Equipment

Centrifugal filter concentrators were from Amicon-Millipore. TosoHaas TSK-gel G3000SWXL gel filtration column was from Hichrom, UK. LAS-1000-3000 charged-coupled device (CCD) Imaging system was from Raytek Scientific. Polypropylene 96-well round bottom, clear plates were from Greiner. 96-well black, optical bottom plates were from Nunc. SpectraMax M2e microplate reader was from Molecular Devices. 5mL Ni-NTA His-trap columns were from GE Healthcare. NanoPhotometer was from Implen. Cell disruptor was from Constant Systems. iBlot Nitrocellulose membranes and iBlot™ Dry Gel Transfer Device were from Invitrogen. Tetra-detector was from Viscotek.

### Strain and plasmid

*S. cerevisiae* W303.1b/GAL4-2 (a, leu2, his3, trp1::TRP1-GAL10-Gal4, ura3, ade2-1, can ^R^, cir+) strain and *pYeDP60*-SERCA1a-BAD expression vector (Amp^R^, ura, ade, OriBact, thrombin cleavage site, Biotin Acceptor Domain) were a gift from Dr Christine Jaxel (Institut de Biologie et de Technologies de Saclay, France) as previously described [17]. *S. cerevisiae* FGY217 (MATa, ura3-52, lys2Δ201, pep4Δ) strain and expression plasmid *pRS426-Gal1-GFP* (ura3, Gal1, Sma1, 8His, yEGFP, Amp^R^) were a gift from Dr. Konstantinos Beis (Membrane Protein Laboratory, Imperial College, London) previously described in [33].

### Cloning human SERCA2a

*pYeDP60*-SERCA1a-BAD vector was linearised using *EcoR1* and *Sma1* restriction enzymes, removing SERCA1a gene. *h*SERCA2a gene, previously mutated for an *EcoR1* restriction site, was cloned into the *pYeDP60*-BAD vector by standard T4DNA ligation and transformed into *E. coli*. The clones were tested by colony PCR and DNA sequencing. *pYeDP60-hSERCA2a-BAD* vector was prepared and used for transformation in yeast using a standard protocol [34].

*h*SERCA2a cDNA was amplified using the following primers:

Forward (5’) ATTAGAATTCTAGTATGGAGAACGCGCACACC(3’) and

Reverse (5’) ATTACCCGGGAGCAGCAGTAGATCCTCTTGGAACCAAACCACCTTCCA

GTATTGCAGGTTCCAGGTAG (3’).

*h*SERCA2a gene was cloned into the *pRS426-Gal1-GFP-His*_*8*_ vector by homologous recombination in *S. cerevisiae*. The vector was linearised using *Sma1* enzyme, providing the blunt ends needed for homologous recombination. The *h*SERCA2a PCR product was mixed with the linearised vector and used for direct transformation into *S. cerevisiae* FGY217, as described previously [35].

*h*SERCA2a cDNA was amplified using the following primers:

Forward (5’) A C C C C G G A T T C T A G A A C T A G T G G A T C C C C CATGGAGAACGCGCACACC (3’) and

Reverse (5’) A A A T T G A C C T T G A A A A T T A A A T T T T C C C CCTCCAGTATTGCAGGTTCC (3’) - the underlined 15 base pair sequence corresponds to the blunt ends of the linearised vector.

All buffers and containers used were sterile and freshly prepared. The obtained transformants were platted on minimal media agar plates (0.1% w/v casaminoacids, 0.7% w/v yeast nitrogen base without amino acids, 2% w/v glucose, 2% w/v agar) and grown for 48 hours at 30^°^C. The clones were tested by PCR and DNA sequencing.

### Expression

The expression level of *h*SERCA2a in *S. cerevisiae* was tested using *pYeDP60* and *pRS426* vectors in small scale minimal media cultures (0.1% w/v casaminoacids, 0.7% w/v yeast nitrogen base without amino acids, 2% w/v glucose). Overnight cultures were used to inoculate 10mL minimal media containing 0.1% w/v glucose at a final OD_600_ 0.12. After complete consumption of the glucose, i.e. when reaching OD_600_ of about 0.6, the expression was induced by 2% w/v galactose. Cells were harvested and processed as in [35] and [18]. Western blotting and in-gel-fluorescence (for the GFP-construct) analysis were used to detect *h*SERCA2a expression.

For large scale cultures 2.5 L baffled Tunair flasks were used, each flask containing 1 L media. An overnight *h*SERCA2a minimal media (2% glucose) pre-culture (300 ml) grown at 30^°^C and 280 rpm shaking was used to inoculate 12L of rich media (2% w/v tryptone, 2% w/v yeast extract, 1% w/v glucose, 2.7% v/v ethanol) or minimal media (0.1% w/v casaminoacids, 0.7% w/v yeast nitrogen base without amino acids and 0.1 glucose % w/v) at a starting OD_600_ of 0.12.

Rich media cultures were grown for 36 hours at 30^°^C and 260 rpm shaking. The culture temperature was lowered to 18^°^C and protein expression was induced by addition of 2% w/v galactose, followed by a second induction with 2% w/v galactose after 15 hours. This methodology was based on previous optimisation of yeast recombinant protein expression established for *rabbit* SERCA1a [16,18], which showed that two inductions increased the expression level of the target protein. Cells were harvested 6 hours after final induction in 1 L centrifuge bottles in a Sorvall Evolution RC centrifuge (10 min at 5000g_av_).

Minimal media cultures were grown for approximately 7 hours at 30^°^C until OD_600_ reached 0.6 and were induced with 2% w/v galactose. Cells were harvested 20 hours after induction, as described above. Cell pellets were frozen using liquid nitrogen and stored at -80^°^C [35].

### Fluorescence size exclusion chromatography

The methodology used for fluorescence size exclusion chromatography was as previously described [35].

### Purification

#### Membrane preparation

Pelleted cells were resuspended in 100mL TES buffer/L culture (50 mM Tris-HCl pH 7.5, 1 mM EDTA, 0.6 M sorbitol, 0.1 M KCl) supplemented with protease inhibitor (1 tablet for each 100 ml buffer), 1 mM PMSF and 5 mM β-ME and passed through the cell disruptor (Constant Systems) three times: once at 30 and twice at 35 Kpsi. Unbroken cells and cell debris were removed by centrifugation for 10 minutes at 15000g_av_ using JLA16.250 rotor. Membranes containing *h*SERCA2a were pelleted by ultracentrifugation at 135000g_av_ (41000 rpm) for two hours using the Type 45 Ti rotor in a Beckman Coulter Optima L100XP ultracentrifuge. The membranes were resuspended and washed in presolubilisation buffer (50 mM MOPS pH 7.0, 100 mM KCl, 1 mM CaCl_2_, 20% glycerol, 5 mM β-ME) and pelleted again by ultracentrifugation for 1 hour at 135000g_av_. Membranes were resuspended in 20 mL HEPES buffer/L culture (20 mM HEPES pH 7.5, 0.3 M sucrose, 0.1 mM CaCl_2_), frozen using liquid nitrogen and stored at -80^°^C.

#### Solubilisation

The membranes were thawed and diluted to 10 mg/ml total membrane protein in solubilisation buffer (50 mM MOPS pH 7.0, 100 mM KCl, 1mM CaCl_2_, 20% v/v glycerol, 5 mM β-ME and 1.5:1 w:w ratio DDM: total membrane protein) and mixed for 1 hour at 4^°^C. Unsolubilised material was removed by ultracentrifugation for 45 min at 135000g_av_ in a Type 45 Ti rotor in a Beckman Coulter Optima L100XP ultracentrifuge.

#### Affinity purification

Ni-NTA super-flow or Talon resin was pre-equilibrated with the solubilisation buffer and incubated with the solubilised material; 1 ml resin/1L culture, for one hour at 4^°^C while on a mixer roller, in the presence of 20 or 15 mM imidazole, respectively, for each resin type, adjusting the pH to 7 to minimise unspecific binding. The resin was then poured into a Bio-Rad glass column and left to settle under gravitational force.

Ni-NTA resin was washed with 30x column volumes of buffer (50 mM MOPS pH 7, 100 mM KCl, 1 mM CaCl_2_, 20% glycerol, 5 mM β-Me, 0.5 mg/ml DDM) and 50 mM imidazole. Elution of bound protein was performed with three column volumes of buffer containing 250 mM imidazole. Similarly, Talon resin was washed with 20x column volumes washing buffer and 15 mM imidazole and bound protein was eluted with two column volumes of buffer containing 150 mM imidazole.

The eluted protein fraction was incubated with TEV-His_6_ protease (0.5 mg TEV protease/L culture used for purification) for GFP-His_8_ tag cleavage and left overnight at 4^°^C while dialysing (dialysis membrane cut-off of 12 kDa; dialysis buffer: 50 mM MOPS, pH 7, 100 mM KCl, 1 mM CaCl_2_, 15% v/v glycerol, 0.25 mg/ml DDM). Since imidazole and DTT are not compatible with the His-Trap column, dialysis was performed to remove excess imidazole and DTT, the latter of which is initially present in the TEV protease buffer. The sample was passed twice through a 5 mL Ni-NTA His-Trap column, previously equilibrated with dialysis buffer to remove the cleaved GFP-His_8_ tag and the TEV-His_6_ protease.

Streptavidin-Sepharose affinity purification was performed as described previously [18] [10]. Briefly, solubilised material was mixed overnight at 4^°^C with Streptavidin-Sepharose resin to allow binding of protein *via* the biotinylated tag, at a ratio *h*SERCA2a: slurry resin of 1 mg: 2 mL, where *h*SERCA2a concentration was assumed to be 1% w/w of total membrane protein concentration, based on previous purification of *r*SERCA1a [17] [30]. The resin was washed with a high-salt buffer (50 mM MOPS-Tris pH 7, 1 M KCl, 20% glycerol (v/v), 1 mM CaCl_2_ and 0.5 mg/ml DDM) and the proteins were eluted in low salt buffer (50 mM MOPS-Tris pH 7, 100 mM KCl, 20% glycerol (v/v), 2.5 mM CaCl_2_ and 0.5 mg/ml DDM) after two short incubations with 25U of Thrombin per ml of settled resin used. Thrombin cleavage action was quenched by addition of 1 mM PMSF. The elution was frozen in the presence of 40% glycerol (v/v), using liquid nitrogen and stored at -80^°^C until further use.

#### Size Exclusion Chromatography

Eluted protein from the affinity purification step was concentrated at 4^°^C and 2600g_av_ in an Eppendorf 5804R bench-top centrifuge, Swing-bucket rotor A-4-44, to 500 μl using 50 or 100 kDa cut-off Millipore centrifugal concentrators. The total protein concentration prior to gel filtration was about 3 mg/ml, as determined by A_280_ method. The sample was loaded onto a TosoHaas TSK-gel G3000SWXL GSK gel filtration column, previously equilibrated with gel filtration buffer (50 mM MOPS pH 7, 80 mM KCl, 1 mM CaCl_2_, 1 mM MgCl_2_, 5 mM β-Me, 0.25 mg/ml DDM or 0.5 mg/ml C _12_E_8_). The chromatography was performed at 4^°^C using a flow of 0.3 ml/minute and the elution was collected in 0.5 mL fractions.

### SERCA2a detection

Ca^2+^-ATPase protein presence was monitored by SDS-PAGE separation followed by Coomassie brilliant blue staining, in-gel-fluorescence analysis, and/or Western blotting analysis using specific antibodies directed against *h*SERCA2. Precast 4-12% Tris-Glycine gels were used to detect the presence of *h*SERCA2a-GFP, throughout the purification steps. Samples were mixed in equal volumes with the loading buffer without boiling, which permitted in-gel-fluorescence visualisation of the fusion GFP-tagged protein by measuring excitation at 460 nm and emission at 515 nm. Also, the fluorescence signal of GFP-membrane protein fusion was monitored in solution using a microplate spectrofluorometer using an excitation wavelength of 488 nm, while measuring emission at 512 nm [35].

### Protein concentration estimation

Total membrane protein concentration was determined using a Bio-Rad_DC_ Protein assay, using bovine serum albumin as standard. Purified protein concentration was determined using a NanoDrop Spectrophotometer, measuring the absorbance at 280 nm and using an extinction coefficient at 280 nm of 99945 M^-1^cm^-1^ for *h*SERCA2a; or by SDS-PAGE quantification, using known amounts of native *rabbit* SERCA1a as standard. The software used to quantify the SERCA2a yield was *ImageJ* [36].

### ATPase activity measurement

The functional assay used is based on an ATP-NADH enzyme coupled assay and involved measuring, spectrophotometrically, the decrease of NADH absorbance at 340 nm [37,38], which is related to ATP consumption. The reaction buffer contained 50 mM TES pH 7.5, 100 mM KCl, 7 mM MgCl_2,_ 0.56/0.28 mg/ml DDM/DOPC mix; 5 mM ATP, 1 mM phosphoenolpyruvate (PEP), 0.2 mg/ml lactate dehydrogenase [39], 0.4 mg/ml protein kinase from rabbit (PK), 1 mM NADH, 1.1 mM EGTA. To determine if the activity observed was Ca^2+^-dependent, the assay was performed at different free Ca^2+^ concentrations (0.0073, 0.0164, 0.042, 0.062, 0.089, 0.20, 0.47, 0.74, 1.1, 2.3, 7.1, 19.6, 34.9, 49.3, 98.8 µM and 1.11mM), which were calculated using *http://maxchelator.stanford.edu/CaMgATPEGTA-TS.htm* programme [40]. For each enzymatic reaction, 150 𝜇L of reaction buffer were incubated at 37^°^C and the reaction was triggered by adding 5-10 µg purified protein. The reaction was quenched with EGTA or thapsigargin (TG). The data were analysed using SigmaPlot Systat software. The slope obtained after quenching was subtracted from the slope obtained after addition of the protein to eliminate any contaminant activity. Specific activity was determined over time intervals where the change in absorbance was linear minus any background activity observed after calcium removal or TG inhibition.

## Results and Discussion

### Recombinant expression of hSERCA2a in *S. cerevisiae*

We have successfully achieved expression of *h*SERCA2a in *S. cerevisiae* using two different constructs (Table 1). One construct was designed with a cleavable C-terminal biotin acceptor domain (*h*SERCA2a-BAD) and a second construct with a cleavable C-terminal His-tagged green fluorescence protein (*h*SERCA2a-GFP-His_8_). An estimated expression level of up to 5 mg of solubilised *h*SERCA2a-GFP-His_8_ per litre of culture was obtained, which is comparable to that reported for heterologous expression of *r*SERCA1a isoform using various constructs in different cell lines, including mammalian and *S. cerevisiae* [16–18,41].

**Table 1:** *Human* cardiac SERCA2a protein yield obtained per 1L of culture *S. cerevisiae* using minimal and rich media.

**SERCA construct**	**Total membrane protein (g)**	**Estimated Ca^2+^ ATPase**		
		**Solubilised material (mg)**	**After affinity purification (mg)**	**After SEC (µg)**
**SERCA2a-GFP-His_8_**	1.1	5.0	0.40	100
**SERCA2a-BAD**	1.0	n/d	0.15	65
**SERCA2a-GFP-His_8_ minimal media**	0.2	1.2	0.18	50

The yields were estimated from SDS-Page gels and Western Blots, using ImageJ software [36,70]. The cultures were grown in rich media unless specified otherwise. Data was estimated for each construct on at least two batches of protein sample, using rabbit SERCA1a and free GFP as control standards; n/d- not determined.

**Figure 1:**
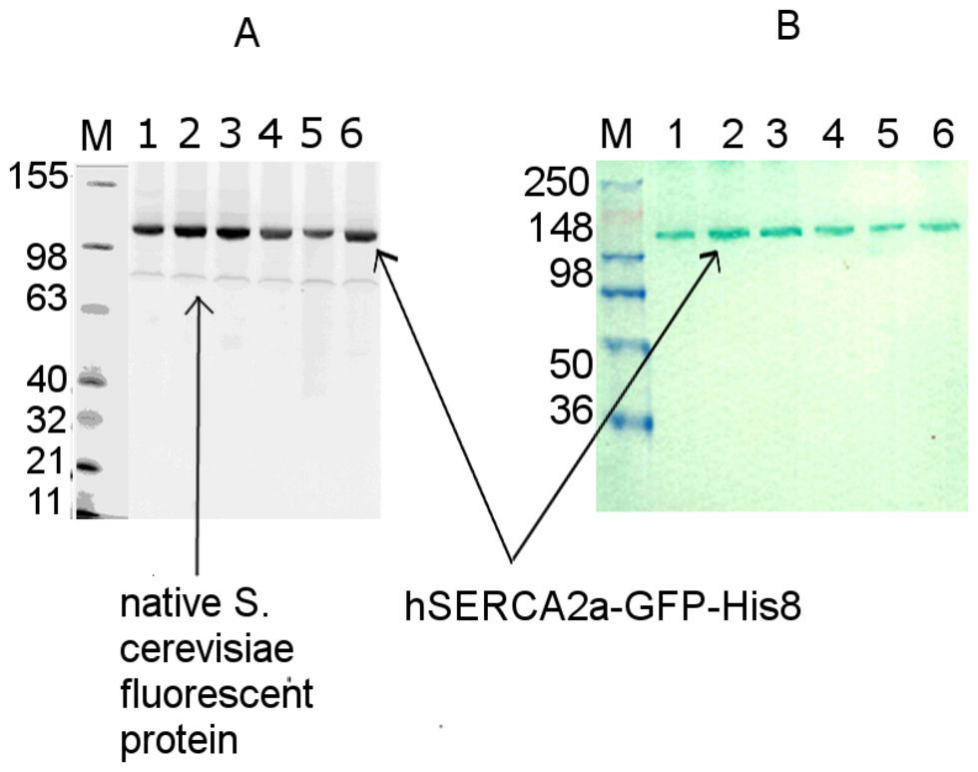
Small scale expression test for *h*SERCA2a-GFP-His_8_ using different clones. **A**. SDS-PAGE 4-12% Tris-Glycine in gel fluorescence analysis. M-fluorescent ladder, 1-6 different *h*SERCA2a-GFP-His_8_ clones; **B**. Western Blotting using specific *h*SERCA2 antibodies, MM – prestained protein ladder.

Given the comparable yields for the two constructs, the *h*SERCA2a-GFP-His_8_ construct offers two main advantages: a) the purification is more cost effective as the resin for affinity chromatography can be re-used and b) the GFP-fusion protein can easily be monitored throughout the expression and purification steps by in-gel fluorescence, with the GFP detection being as sensitive as or better than Western blot detection using a *h*SERCA2 specific antibody. An example of assessment of *h*SERCA2a-GFP-His_8_ expression levels in different clones using in-gel fluorescence and Western blotting techniques is shown in Figure 1.

To ensure the highest possible expression levels of functional *h*SERCA2a-GFP-His_8_, we used rich medium and lowered the temperature to 18^°^C, before two steps of galactose induction of protein expression [16]. In addition, the use of baffled flasks for cell growth significantly improves media aeration and allows routine yields of 50-60 g of yeast per litre culture to be obtained. We also tested *h*SERCA2a-GFP-His_8_ expression levels in minimal medium and determined expression levels above 1mg L^-1^ culture (Table 1) of solubilised protein. Minimal medium cell culture growth led to much lower cell mass yield (<10g L^-1^ culture).

Previous studies have shown that chemical chaperons such as DMSO (2.5% v/v), histidine (0.04% w/v) or biotin (0.2̶2 mg/ml) can improve the expression level of recombinant proteins [33,42]. To further increase *h*SERCA2a expression, we performed small scale tests in the presence of DMSO and histidine for the *h*SERCA2a-GFP-His_8_ construct or biotin for the *h*SERCA2a-Biotin construct. Addition of these chemicals was performed concomitant with galactose induction. No visible difference was observed in the expression levels (data not shown), as previously reported for *r*SERCA1a in the case of biotin addition [8].

### Isolation of hSERCA2a-Biotin

**Figure 2:**
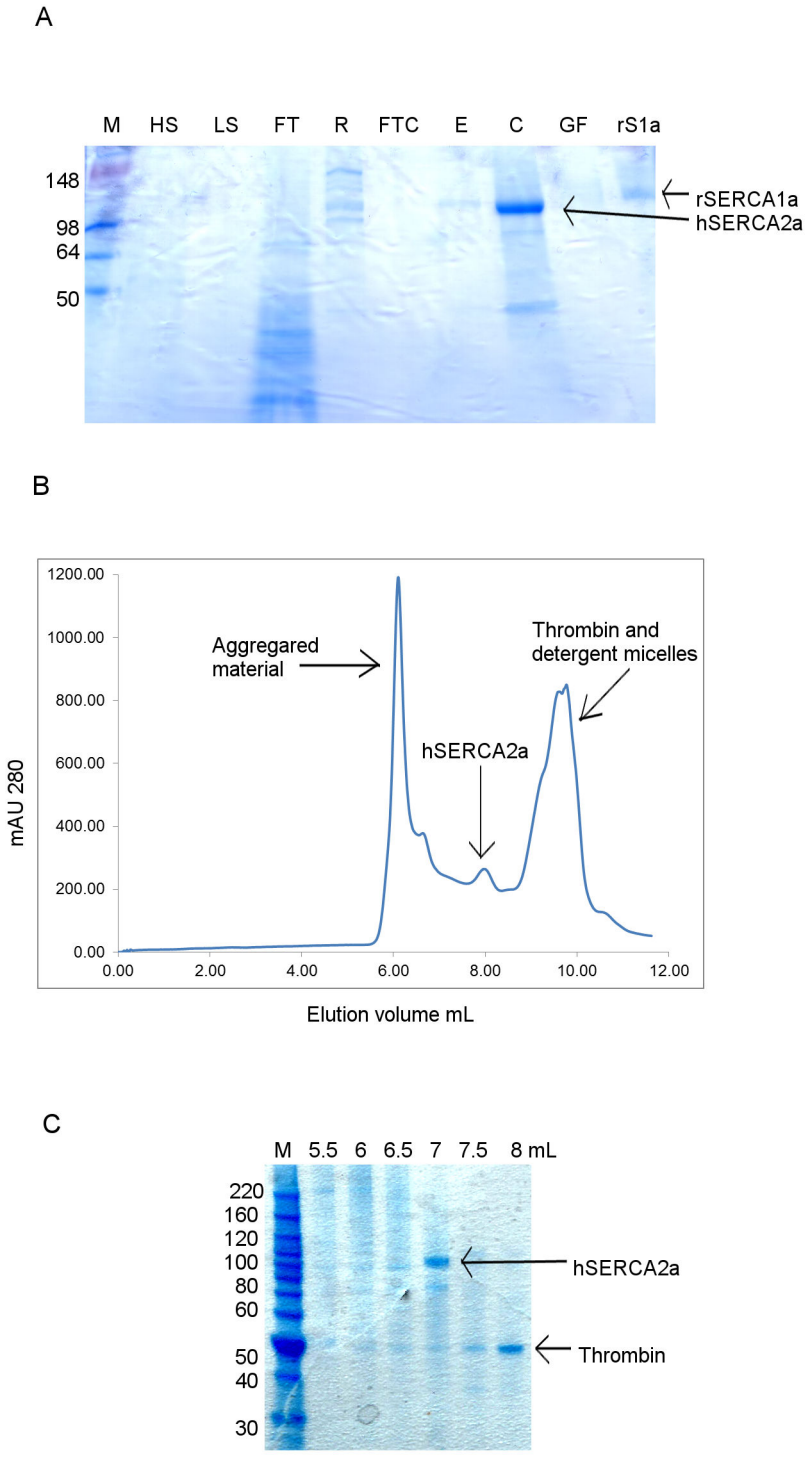
Purification of *h*SERCA2a-Biotin fusion protein using Streptavidin-Sepharose resin. **A**. Coomassie stained 12% Tris-Glycine SDS PAGE gel from purification, M- protein ladder, HS-high salt wash, LS-low salt wash, FT- flow-through after binding to Streptavidin resin, R-resin with bound hSERCA2a, FTC- flow-through concentrated, E – elution from Streptavidin resin, C- concentrated sample prior to gel filtration, GF- elution after gel filtration, rS1a- rabbit SERCA1a, 1µg. **B**. HPLC-SEC profile for hSERCA2a after Streptavidin affinity purification using 12L culture. DDM detergent was exchanged on gel filtration column with C_12_E_8_ detergent. **C**. Coomassie stained 4-12% Bis-Tris SDS PAGE gel. SEC fractions obtained for *h*SERCA2a purified with Streptavidin resin.

Purification of *h*SERCA2a-Biotin, using its biotinylated tag, produced pure and functionally active protein. The resulting purified protein is shown in Figure 2. Yields of approximately 150 µg of purified *h*SERCA2a per litre culture were obtained after affinity chromatography and 65 µg per litre after size exclusion chromatography (SEC), Table 1. This is slightly lower compared to the yield obtained for *r*SERCA1a, using the same approach [18], and comparable to the yield obtained for purified *h*SERCA2a by Magro et al. [43], using surface active maghemite nanoparticles for purification (65 𝜇g/L at >90% purity versus 125 𝜇g/L at 70% purity, respectively). However, the *h*SERCA2a protein obtained in this study is nearly pure (> 90%) following SEC (Figure 2C) and is shown to be functionally active.

The biotin-fusion protein purification methodology required the use of new Streptavidin-Sepharose resin for each purification, due to the strong interaction of biotin with streptavidin resin. Thus, alternative approaches to express and purify *h*SERCA2a from *S. cerevisiae* were investigated in order to reduce the cost of purification. Immobilized metal affinity chromatography (IMAC) purification is more economical and can be useful for membrane protein purification [44,45]. However, a GFP-His fusion tag additionally offers the ability to follow easily the over-expressed protein during the expression and purification steps [35].

### Isolation of hSERCA2a-GFP-His_8_

**Figure 3:**
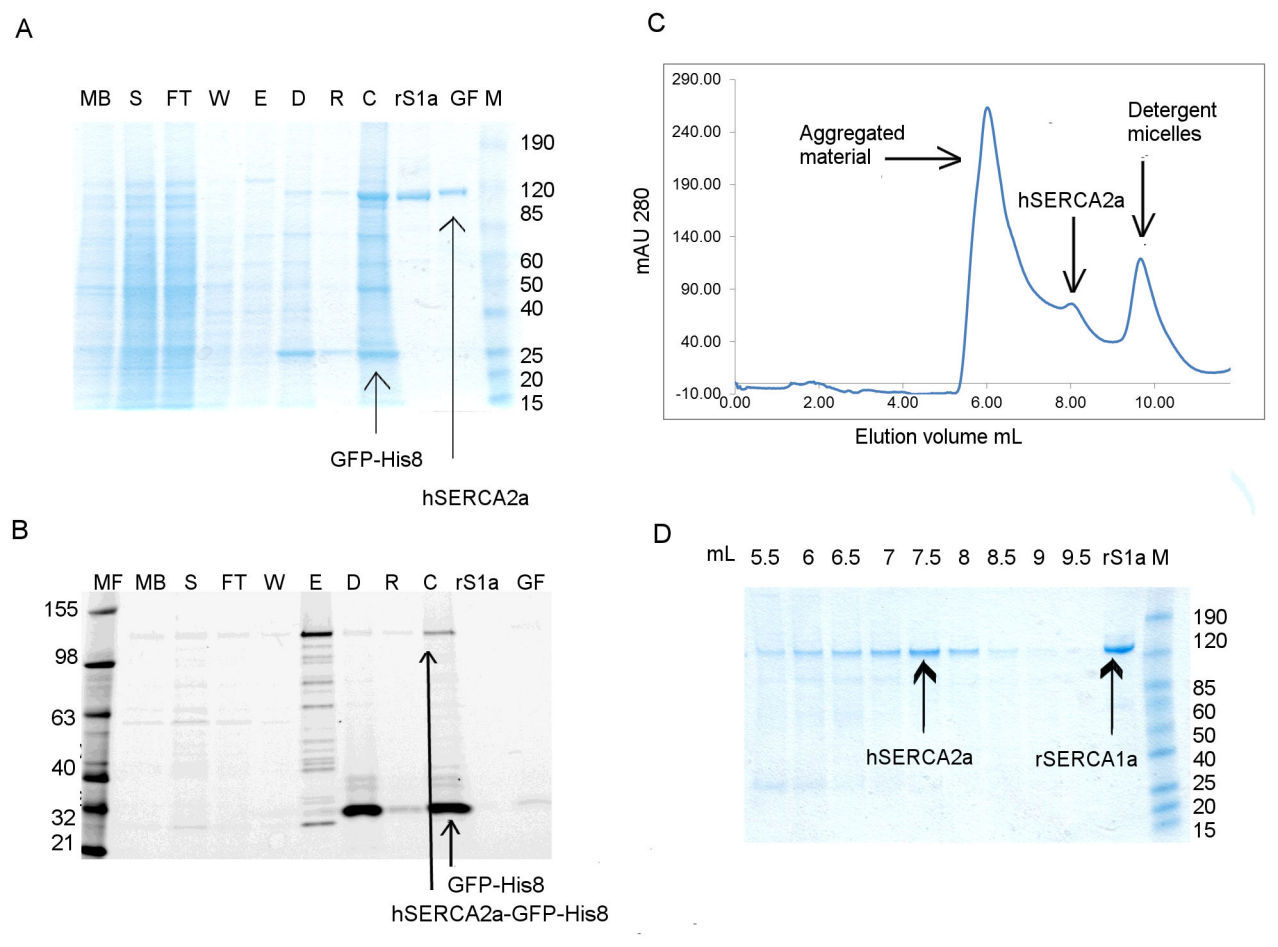
Purification of *h*SERCA2a-GFP-His_8_ using Ni-NTA affinity chromatography. Purification was done in the presence of DDM only throughout all steps, including SEC. Protein was obtained using rich media. **A**. Coomassie stained SDS-PAGE gel; **B**. In gel fluorescence 12% Tris-Glycine SDS-PAGE gel. MF- fluorescent protein ladder; MB- diluted membrane fraction; S- solubilised fraction; FT- flow-through after binding; W- wash fraction; E- elution; D- sample after cleavage with TEV protease and dialysis; R- sample after Ni-NTA rebinding after tag cleavage; C- sample concentrated using 50 kDa cut-off filter concentrator, before gel filtration; S1a- rabbit SERCA1a; GF- fraction containing *human* SERCA2a after gel filtration; M- prestained protein ladder. **C**. HPLC-SEC profile for *h*SERCA2a purified using Ni-NTA super-flow resin. **D**. Coomassie stained 4-12% Tris-Glycine SDS PAGE gel for SEC fractions obtained for purification of *h*SERCA2a using Ni-NTA super-flow resin.

Purification of *h*SERCA2a-GFP-His_8_ was performed initially using Ni-NTA batch-mode binding [35]. The yield of *h*SERCA2a after affinity purification was improved to 0.4 mg per litre of culture (Table 1). Despite the higher yield, more than half of the solubilised material is not bound to the resin but is recovered in the flow-through after the affinity step, as observed using in-gel fluorescence (Figure 3B, lane FT versus S). Further, poor separation of the monomer and oligomerised/aggregated fraction during size exclusion chromatography was observed (Figure 3C). Oligomers are expected when SEC is performed in the presence of C_12_E_8_ [11]. Note that the contribution of light scattering in OD for protein aggregates is high but in fact it corresponds to a relatively low amount of protein, as demonstrated in Figure 3D (see lane 6.0 and 6.5 mL). However, the protein following SEC chromatography is almost pure, as shown by Coomassie Blue stained gel (Figure 3A, lane GF). Purification of *h*SERCA2a-GFP-His_8_ using minimal medium yielded approximately 180 µg of purified *h*SERCA2a per litre culture, after affinity purification (Table 1). This is less than half compared to the yield when using rich medium. The benefit of using minimal medium, besides being selective for SERCA2 vector containing cells, is that it provides a reduced amount of membranes at the start of the purification, thus requiring less detergent for solubilisation than when using rich medium. The final yield of *h*SERCA2a after SEC, obtained using minimal media, is approximately 50 µg per litre of culture, which means a lower yield per litre culture than when using rich media but a higher yield per wet cell weight. Also, minimal media resulted in a lower amount of total membrane protein loaded on the affinity resin, which can be important to reduce the amount of unspecific bound material.

### Optimising the purification of hSERCA2a-GFP-His_8_

**Figure 4:**
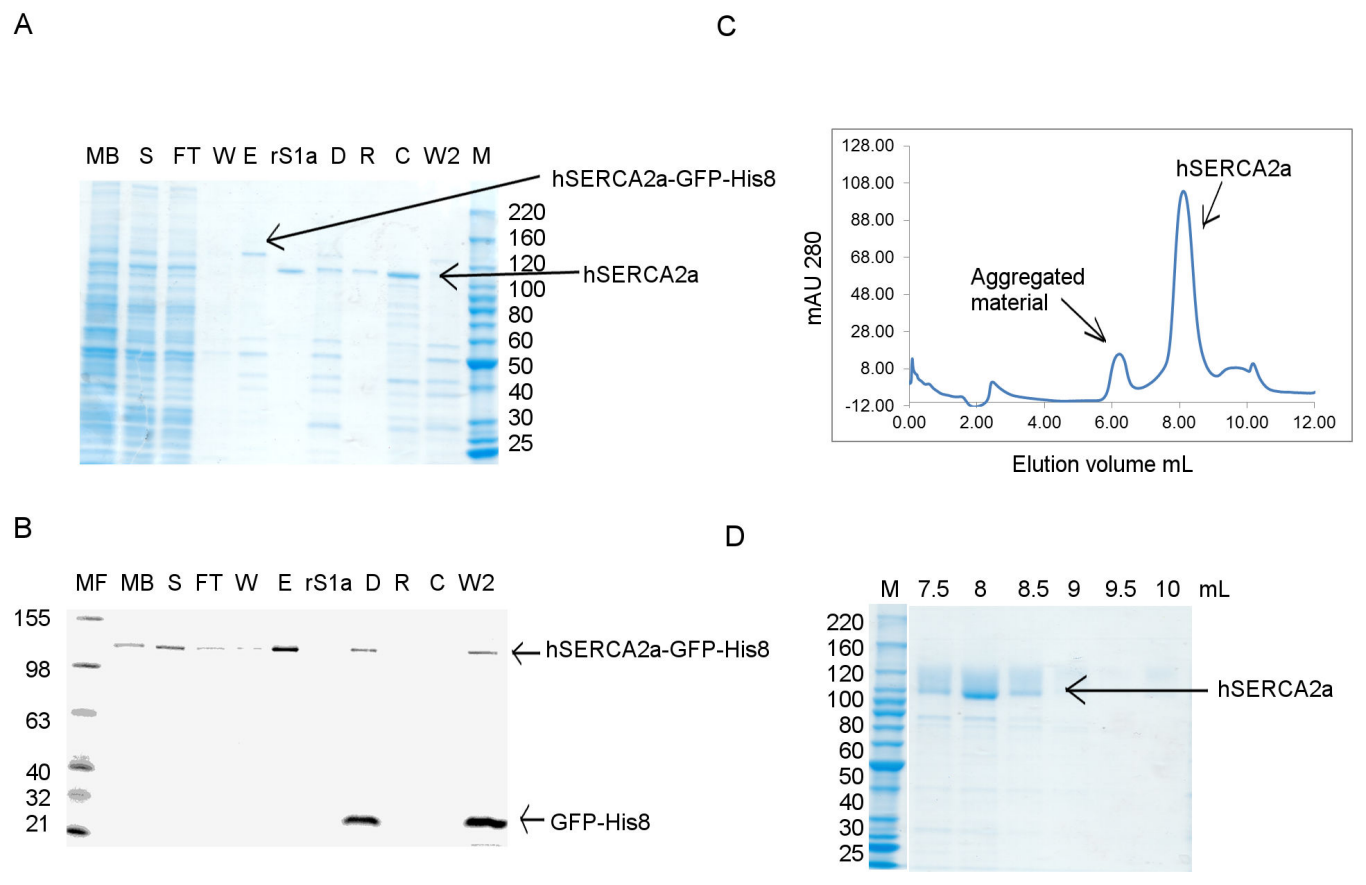
Talon resin affinity purification of *h*SERCA2a-GFP-His_8_. Protein was obtained using minimal media. **A**. Coomassie stained SDS-PAGE gel; **B**. In gel fluorescence 12% Tris-Glycine SDS-PAGE gel, MF- fluorescent protein ladder; MB- membranes fraction; S- solubilised fraction; FT- flow-through after binding to Talon resin; W- wash fraction; E- elution; D- sample after cleavage with TEV protease and dialysis; R- sample after Ni-NTA reverse binding; C- sample concentrated, before gel filtration; rS1a- rabbit SERCA1a at; C- fraction containing *human* SERCA2a concentrated before gel filtration; W2- eluted material from Ni-NTA His trap after reverse Ni-NTA purification; M- protein ladder. **C**. HPLC-SEC profile for *h*SERCA2a purified using Talon resin. Protein was concentrated with 100 kDa cut-off filter concentrator and DDM detergent was exchanged on gel filtration column with C_12_E_8_ detergent. **D**. Coomassie stained 4-12% Tris-Glycine SDS PAGE gel for SEC fractions obtained for purification of *h*SERCA2a using Talon super-flow resin.

After several optimisation tests (using higher salt concentration in purification buffers and varying the detergent concentration used during solubilisation), Talon resin was used in an attempt to improve the yield during the affinity purification step (Figure 4). Talon resin is an IMAC resin, similar to Ni-NTA, but charged with cobalt ions instead of nickel. It is described to bind His-tagged proteins with higher specificity than nickel-charged resins, resulting in isolation of His-tagged proteins with higher purity and lower metal ion leakage (Co^2+^ ions are more tightly bound to NTA than Ni^2+^ ions) [46]. As anticipated, the Talon resin proved to be more specific than the Ni-NTA resin. Binding at 15 mM imidazole and at pH 7 was essential for obtaining a cleaner fraction, as binding at 5 or 10 mM imidazole led to higher unspecific binding. Following TEV-His_6_ protease cleavage, the protein was passed twice through the Ni-NTA His trap column to remove uncleaved material and any contaminants bound to the affinity resin. We observed that passing the sample twice, rather than once, through the Ni-NTA column improved the purity of the sample (Figure S1A, lane R1 and R2). The yield of purified protein, for the *h*SERCA2a-GFP-His_8_ construct, following the second purification step (SEC-HPLC), was 100 µg *h*SERCA2a per litre of culture (Table 1).

Purification of *h*SERCA2 tagged only with the deca-histidine (-His_10_) tag rather than the GFP-octa-histidine (-GFP-His_8_) tag was also tried, but no improvement was observed in the purification profile during size exclusion chromatography (Figure S2), suggesting no effect of the size of the histidine tag or of the presence of GFP on aggregation.

The main difficulty with the purification of *h*SERCA2a-GFP-His_8_, using Ni-NTA resin, is the low level of binding of the solubilised protein to the metal-affinity matrix. This was observed by in-gel fluorescence analysis of the flow-through sample after binding (Figure 3B). No improvement was observed even when performing a longer binding step or when rebinding of unbound material was tried (data not shown). There are several potential factors contributing to the low recovery observed. A fraction of the expressed protein may not be correctly inserted into the membrane and hence misfolded to some extent, resulting in shielding the GFP-His-tag. Alternatively, the GFP-His-tag may only be partially accessible without any misfolding of the fusion protein, as suggested in a comparable study [13]. The latter hypothesis is sustained by our current results, such that high fluorescence intensity of GFP-fusion protein (Figure 3B) indicates correct folding of the protein, in agreement with previous work using GFP [33,35,47]. However, this does not eliminate the possibility of partial unfolding of the protein during the extraction from the membranes. From this perspective, any or all of these factors may lead to reduced recovery. One might argue that the poor binding of *h*SERCA2a-GFP-His_8_ may be due to low binding affinity of the fusion protein for the metal resin. Nevertheless, a step gradient elution of GFP tagged protein from Ni-NTA resin revealed that the protein starts to elute at and above 75 mM imidazole, indicating strong binding and proper folding of the expressed protein. In this respect, the poor affinity could be considered an advantage as it allows us to eliminate misfolded and probably inactive enzymes leading to optimal purification of properly folded *h*SERCA2a-GFP-His_8_.

### Aggregation properties and effect on purification

To address the possibility that aggregation of *h*SERCA2a-GFP-His_8_ was induced by the solubilisation procedure, various DDM: total membrane protein concentration ratios were tried. We observed that lowering the ratio from 3:1 to 1:1 does not affect the recovery of *h*SERCA2a-GFP-His_8_ during the affinity chromatography step. Fluorescence size exclusion chromatography (FSEC) analysis [48] was used to investigate whether using DDM, C_12_E_8_ or a lipid-like detergent ,FC12, in the absence or presence of cholesterol hemissuccinate salt could improve the yield of recovered protein. FC12 detergent and cholesterol have been shown to improve the solubility and stability of heterologous membrane proteins [35]. FSEC analysis showed that *h*SERCA2a-GFP-His_8_ solubilisation in the presence of FC12 and cholesterol presented the highest monodispersity (FigureS3A), although further large scale purification revealed that this detergent-lipid combination led to aggregates after solubilisation without any improvement in the yield of monodispersed sample after SEC (Figure S3B). This may be due to the high solubility capacity of the FC12 detergent.

The final purification step for both constructs (-Biotin and -GFP-His_8_) is size exclusion chromatography with detergent exchange from DDM to C_12_E_8_. Detergent exchange has been shown to be critical for native and recombinant SERCA1a crystallisation [15,49,50]. Figure 4C shows a typical SEC profile for *h*SERCA2a-GFP-His_8_ after purification, highlighting the two different populations observed i.e. an aggregated fraction (at ~5.5-6.5 mL) and a monomeric fraction (at ~7.5-8.0 mL). For all GFP-His_8_ purified samples we observed a third peak in the chromatograms (Figure 3C, from ~9.0–10.5 mL), which was assigned to correspond to detergent micelles [51] since it did not appear to contain protein, as observed by SDS-PAGE (Figure 3D). To further investigate the identity of this third peak, we used a Tetra-detector, which allows measurement of refractive index by light scattering of membrane protein in detergent solution [52]. The sensitivity of this method can reveal the presence of free detergent micelles and the ratio between protein and detergent present in the sample [51,53]. For comparison, we analysed the sample before SEC (concentrated using 50kDa cut-off filter concentrators) and the fraction corresponding to the monomeric fraction, after gel filtration. The refractive index peak corresponding to the detergent micelles is present only in the sample before SEC (Figure S4A) and not in the sample corresponding to the monomeric *h*SERCA2a (Figure S4B). This suggests that most of the detergent micelles were eliminated during the SEC step. We also observed that using 100 kDa cut-off concentrators instead of 50 kDa cut-off removes free detergent micelles from solution (Figure 4C), as indicated by refractive index measurement, at the expense of 12% protein loss as previously shown in similar experiments [54]. This point is critical, as final detergent concentrations need to be tightly controlled for crystallisation trials [49].

### Enzymatic properties of the purified hSERCA2a

To characterise the functional properties of the purified *h*SERCA2a, the enzymatic activity was measured using a regenerative ATP-NADH enzyme coupled assay [38,55]. For all functional assays, only SEC-HPLC purified protein, which had previously undergone all purification steps (including cleavage of any affinity tag), was tested.

**Figure 5:**
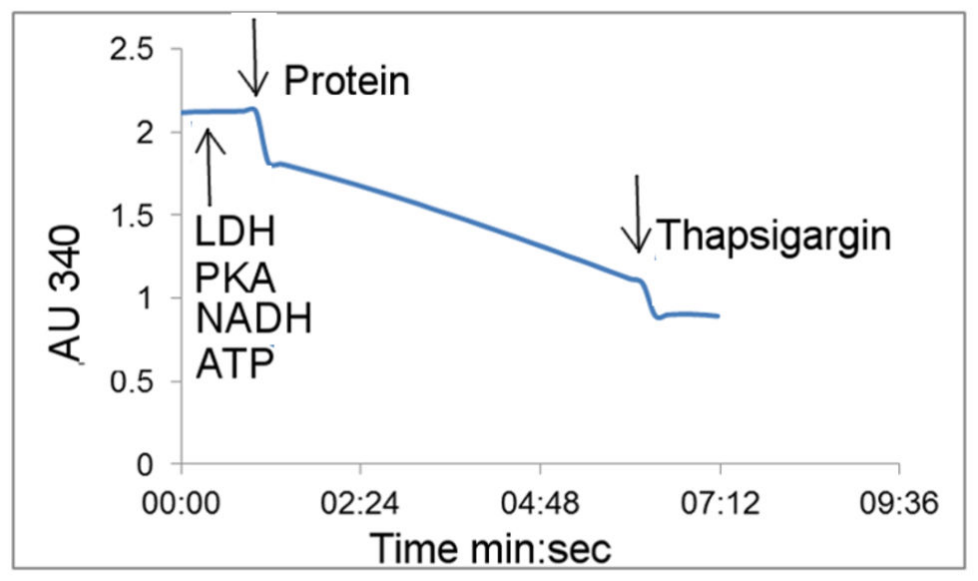
Typical activity assay profile for purified recombinant *human* Ca^2+^ ATPase isoform 2a (*h*SERCA2a). The protein was purified using -GFP-His_8_ tag and Talon resin affinity purification. Reaction buffer used was as in Materials and Methods; the reaction was triggered by adding 5 µg of purified protein. Addition of thapsigargin inhibits activity of purified protein. Calcium-dependent activity corresponds to the difference of slope before and after thapsigargin addition. Here, final calcium-dependent ATPase activity is about 3 µmol hydrolysed ATP/min/mg of *h*SERCA2a.

A typical spectroscopic trace, measuring NADH absorbance at 340 nm, is shown in Figure 5. Upon addition of *h*SERCA2a to the reaction mixture, a linear reduction in absorbance is observed indicative of NADH conversion to NAD^+^ as a consequence of ATP hydrolysis by the purified *h*SERCA2a. As expected, after addition of molar equivalent concentrations of EGTA (equivalent to the free Ca^2+^ concentration) or thapsigargin (equivalent to the protein concentration), ATP hydrolysis is quenched, confirming the presence of a calcium and thapsigargin dependent *h*SERCA2a.

### Specific enzymatic activity

ATPase calcium dependent analysis of *h*SERCA2a (from *h*SERCA2a-GFP-His_8_) revealed a variation in the specific enzymatic activity from 1 to 3 µmol min^-1^ mg^-1^ protein between different batches of purified protein. However, we observed that *h*SERCA2a, solubilised and purified in the presence of DDM, consistently showed lower specific activity compared to *h*SERCA2a solubilised in the presence of DDM and exchanged for C_12_E_8,_ during the final SEC purification step. The presence of low amounts of DDM monomers, close to the Ca^2+^ translocating protein, may affect its enzymatic activity as demonstrated in previous studies on *rabbit* SERCA1a [56]. Thus, exchange of DDM for C_12_E_8_ during SEC purification is the most efficient way to remove DDM from the ATPase monomers. Another reason for the observed variability in specific activity may be the measurement of protein concentration in the eluted fractions, which is difficult to assess considering the low concentration.

Previously published data showed an enzymatic ATPase turnover value of 70 sec^-1^ for *h*SERCA2a, when expressed in HEK cells [57], 30 sec^-1^ when expressed in COS cells [3] and 35 sec^-1^ when obtained from natural source [3]. Our results have shown a turn-over rate of 2-5 sec^-1^, depending on the protein batch used. Assuming there is no inactive protein after purification, which is reasonably difficult to assess, the differences in ATPase turn-over rate between *h*SERCA2a expressed in HEK, COS cells, or retrieved from natural source and the values obtained in the present study could arise from the difference in activity between membrane embedded and detergent solubilised SERCA2a. Interestingly, these differences are not observed for SERCA1a when purified from natural source [58] or after heterologous expression [59,60]. The natural environment surrounding *h*SERCA2a is very different from the surrounding environment in this study. The major lipid found in heart tissue is phosphatidylcholine followed by phosphatidylethanolamine [61,62]. Exactly what part of *h*SERCA2a is embedded in the lipid bilayer is not known [63] and it is not clear to what extent it differs from the part embedded into a detergent micelle after solubilisation. Detergent solubilisation and extensive chromatography steps delipidate the protein and may cause a destabilising effect. In some cases, detergent purified *r*SERCA1a presents a higher activity upon relipidation (DOPC) [56,64]. Thus, the interactions between the lipids and *h*SERCA2a may be important for the protein to demonstrate maximum turnover [65].

### Ca^2+^-dependent ATP hydrolysis

**Figure 6:**
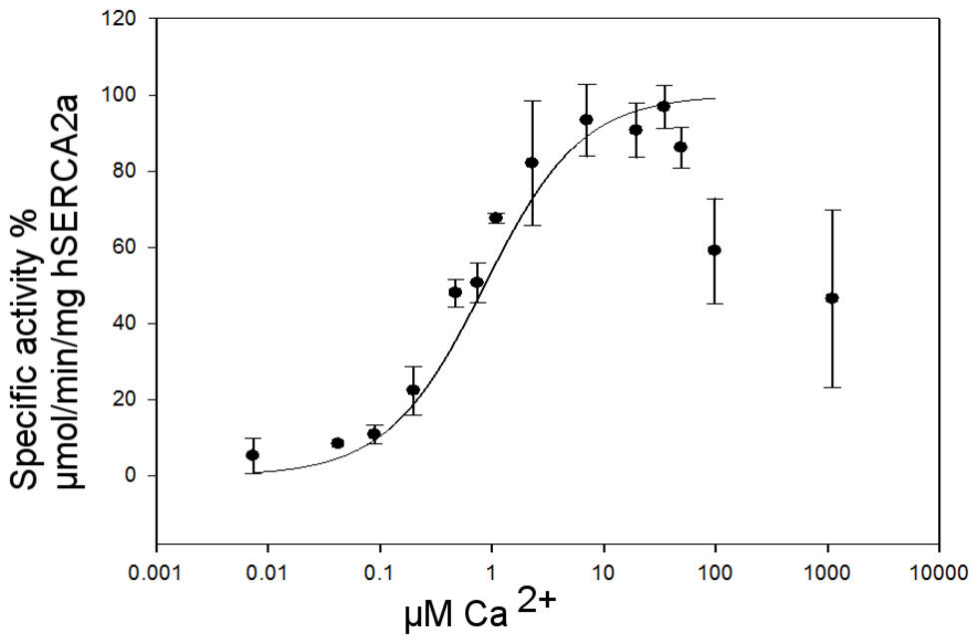
Normalised specific ATPase activity rate versus Ca^2+^ dependence for DDM solubilised *h*SERCA2a after HPLC-SEC purification. 100% specific ATPase activity corresponds to 3 µmol hydrolysed ATP/min/mg of *h*SERCA2a. The results are the means of seven measurements, using protein obtained from three independent membrane preparations; error bars represent ±S.D.

Calcium-dependence of the ATP hydrolysis using an ATP-NADH coupled assay was estimated as described above. As shown in Figure 6, we observed calcium dependent activation of *h*SERCA2a from which an apparent calcium affinity of approximately 0.6 μM for DDM-solubilised *h*SERCA2a was determined. This value is in agreement with previously published values for apparent calcium affinity, K_0.5_ values ranging from 0.2 to 0.9 μM [3,22,57,66,67], depending on the nature of the protein sample. There is a notable sharp decrease of activity at calcium concentrations higher than 100 µM. As observed for *r*SERCA1a, high calcium concentrations inhibit the pump due to: i) calcium affinity for the luminal binding sites is in the mM range and binding of calcium on the luminal side dramatically slows down the dephosphorylation step, and ii) Ca^2+^ATP can replace Mg^2+^ATP at the nucleotide binding site, resulting in deceleration of the phosphorylation step.

## Conclusions

We have succeeded in expressing and purifying functionally active, recombinant *h*SERCA2a using *S. cerevisiae* and we used the purified enzyme to characterise its functional properties. The *h*SERCA2a yield reported in this study, using the biotinylated tag, is comparable to that observed by Magro et al. [43]. Furthermore, our work has demonstrated a purification protocol for functionally active *h*SERCA2a.

The differences in purification yield between that previously reported for *rabbit* SERCA1a [18] and *h*SERCA2a obtained during this work using *S. cerevisiae*, may be explained by the differences in the amino-acid sequences of the two proteins, particularly those present in the C-terminal tail region. It would be interesting to estimate the level of *in vivo* biotinylation for the *h*SERCA2a-Biotin, which could reveal the amount of properly folded protein [17,50].

Alternatively, the differences in purification yield observed could be due to the particular post-translational modifications of *h*SERCA2a, as recent heart failure research revealed that SUMOylation by SUMO-1 of *h*SERCA2a contributes to its stability [68]. It may be that the SUMO-like machinery present in *S. cerevisiae* is not completely equivalent to that in *human* [69]. The SUMOylation modification of SERCA2a could be potentially sustained in yeast through the SUMO-1 homolog SMT3 (suppressor of mif two 3 (macrophage migration inhibitory factor, glycosylation-inhibiting factor)). However, currently there are no published data available to confirm that SUMOlylation of the heterologous expressed protein takes place. Future work could involve Western blotting analysis of heterologously expressed *h*SERCA2a proteins with double-labelling against SERCA proteins and SMT3 to check for SUMOylation in yeast. If no SUMOylation is found, then a heterologous co-expression of SUMO-1 and SERCA2 should be considered.

With regard to the solubilisation detergent, as tested in the case of the *h*SERCA2a-GFP-His_8_ construct, no increase in the yield of purified protein was observed when using a smaller detergent: total membrane protein ratio. Thus, it can be concluded that the initial 3:1 ratio used did not contribute to the high aggregation observed during SEC. Further, based on the result obtained with FC12, it may be that usage of a higher solubility capacity detergent may lead to aggregated fraction solubilisation rather than the solubilisation of the active form of the protein. Further work would be necessary to more fully understand the effect of different detergents or lipids on *human* cardiac Ca^2+^-ATPase stability [58].

Purified *h*SERCA2a (from *h*SERCA2a-GFP-His_8_ construct) showed calcium-dependent and thapsigargin-sensitive activity. The calcium K_0.5_ for *h*SERCA2a of 0.6 μM found herein is within previously reported values [57]. A significant difference observed in the turn-over rate between previously purified samples of SERCA2a may be explained by the different lipid content, since previous data were measured on vesicular microsomes which contain some natural lipids associated with SERCA2, whereas SERCA2a purified from *S. cerevisiae* was not reconstituted into liposomes and was analysed in the presence of detergent. Another plausible explanation for the differences in turn-over rate could be the presence of inactive protein in the final purified sample. Finally and importantly, are the diversity of ATPase assay conditions (e.g. functional assay, temperature, pH, buffer composition), which may explain the significant differences in the enzymatic activities reported. It is noteworthy that, although the turn-over rate for *h*SERCA2a was different than when expressed using other systems (HEK, COS, natural source), the values obtained here are very close to the specific enzymatic activities obtained for *r*SERCA1a expressed and purified from *S. cerevisiae* [16,17].

The optimised protocol outlined in this work is easily extended to other SERCA isoforms and useful for the production of high quality recombinant active protein for further analysis to study interactions between SERCAs and their physiologically relevant partners. The resulting protein is suitable for crystallisation trials and subsequent structural analysis. Furthermore, the method outlined may prove useful generally for the recombinant production of other multi-domain eukaryotic membrane proteins.

## Supporting Information

Figure S1 **Purification optimisation: two Ni-NTA reverse binding steps for *h*SERCA2a-GFP-His_8_.** Coomassie stained 4-12% Tris-Glycine SDS PAGE gel: M- protein ladder, E- eluted protein, D- sample after cleavage with TEV protease and dialysis, R1- after first Ni-NTA reverse binding, R2- after second Ni-NTA reverse binding.
(TIF)

Figure S2 **Comparison of SEC purification profile of *h*SERCA2a-His_10_ and *h*SERCA2a-GFP-His_8_.** **A**. SEC profile for *h*SERCA2a-His_10_. **B**. SEC profile for *h*SERCA-GFP-His_8_. Affinity purification was performed using Talon resin. The purification protocol did not involve washing the membranes prior to solubilisation. The protein was concentrated with 50KDa cut-off filter concentrators prior to SEC purification.
(TIF)

Figure S3 **Detergent screening for *h*SERCA2a-GFP-His_8_.** **A**. Fluorescence size exclusion chromatography profile for *h*SERCA2a-GFP-His_8_ solubilised in the presence of 2% w/v cholesterol hemisuccinate salt and 1% w/v FC12 or 1% w/v β-DDM. The same volume of solubilised *h*SERCA2-GFP-His_8_ was loaded onto a Superose 6 10/300 column for each detergent screen. The eluted fractions were analysed using a SpectraMax spectrophotometer, as described in Materials and Methods. Aggregation peak corresponds to the void volume. **B**. HPLC-SEC profile for large scale purification of hSERCA2a-GFP-His, in the presence of FC12 and hemisuccinate salt cholesterol.
(TIF)

Figure S4 **Tetra-detector analysis of purified *h*SERCA2a before and after SEC.** The construct used for this analysis was *h*SERCA2a-GFP-His_8_. **A**. Protein sample analysis before SEC. The profile obtained is comparable to the SEC profiles obtained for *h*SERCA2a. The data shows two UV peaks after the void volume, the first peak (at 13.83mL) corresponds to *h*SERCA2a monomer and the second peak (at 16.31mL) presence a refractive index trace, indicating excess detergent micelles. **B**. Protein sample analysis after SEC. The protein fraction eluted from SEC, corresponding to the monomer form of *h*SERCA2a, was run on the Tetra-detector. The result indicates that the concentrated detergent micelles are separated and eliminated during SEC. The refractive index peak corresponds to the protein-detergent complex.
(TIF)
