# Supplementary figures and images for: Optimisation of Recombinant Production of Active Human Cardiac SERCA2a ATPase
Source: PLoS One. 2013 Aug 12;8(8):e71842. doi: 10.1371/journal.pone.0071842 (PMC3741278; doi:10.1371/journal.pone.0071842)

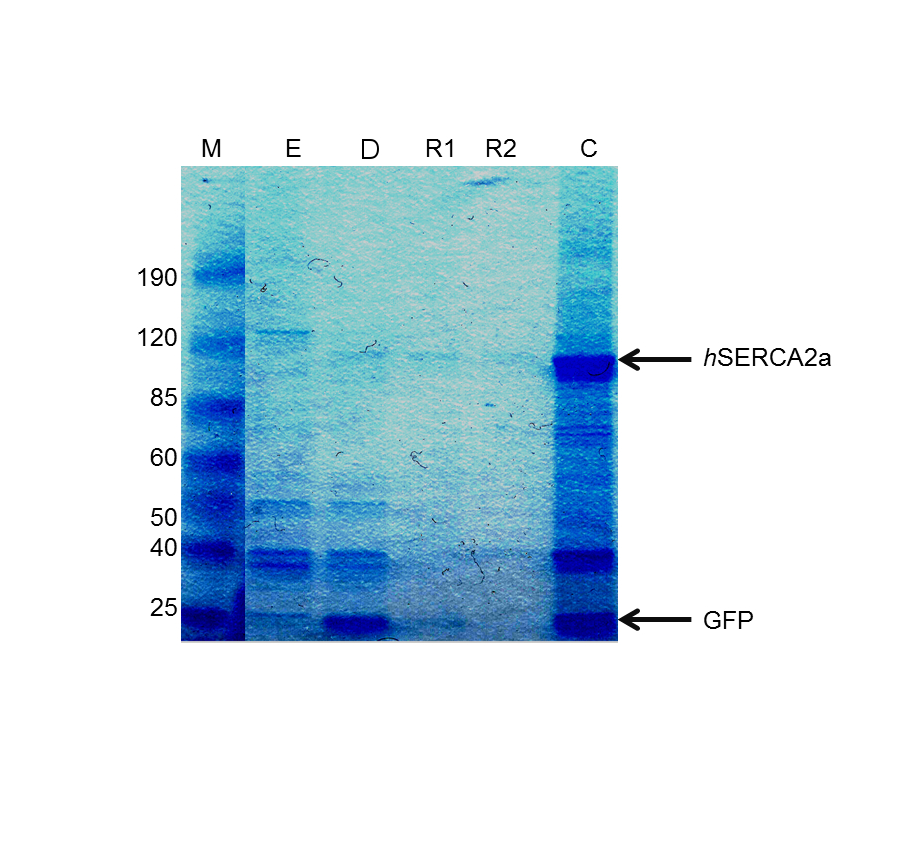

Supplement: Figure S1 — Purification optimisation: two Ni-NTA reverse binding steps for hSERCA2a-GFP-His8. Coomassie stained 4-12% Tris-Glycine SDS PAGE gel: M- protein ladder, E- eluted protein, D- sample after cleavage with TEV protease and dialysis, R1- after first Ni-NTA reverse binding, R2- after second Ni-NTA reverse binding. (TIF) [file pone.0071842.s001.tif]

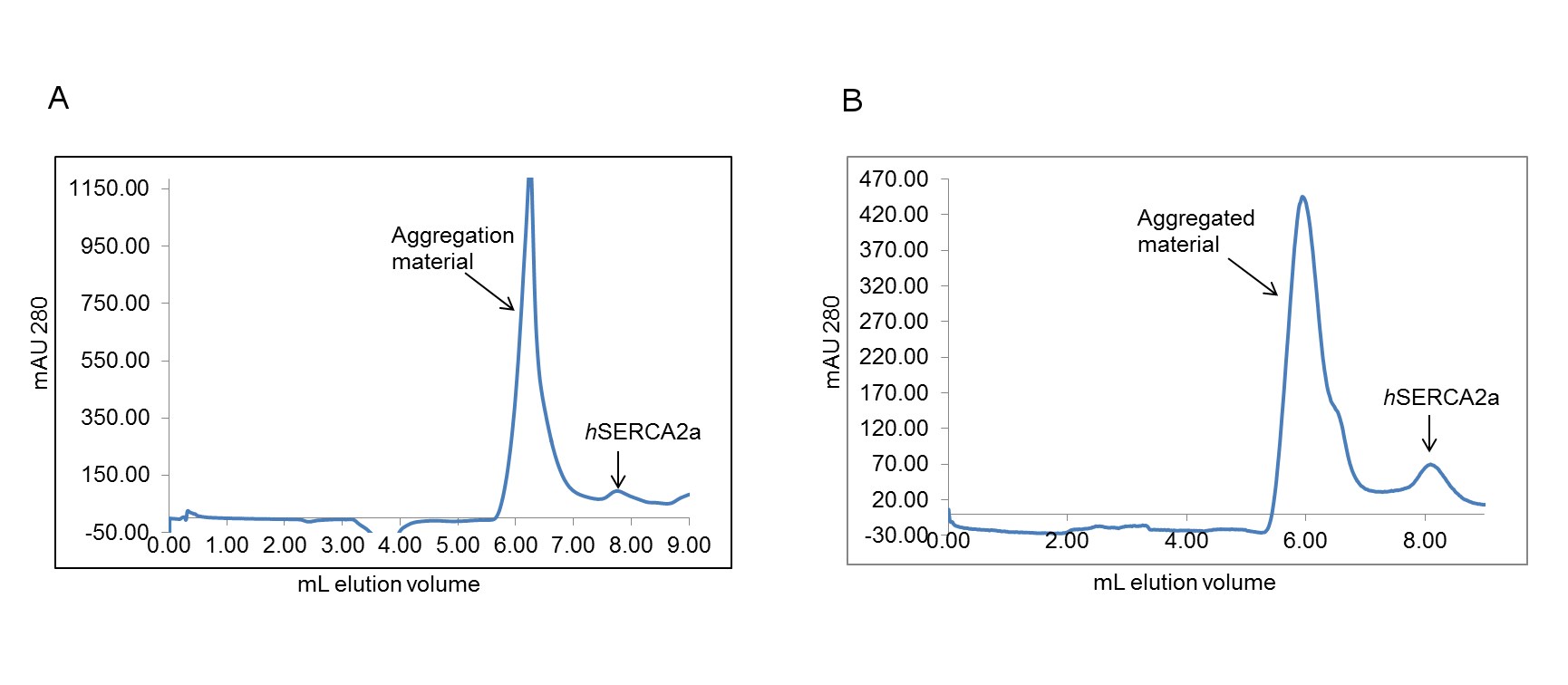

Supplement: Figure S2 — Comparison of SEC purification profile of hSERCA2a-His10 and hSERCA2a-GFP-His8. A. SEC profile for hSERCA2a-His10. B. SEC profile for hSERCA-GFP-His8. Affinity purification was performed using Talon resin. The purification protocol did not involve washing the membranes prior to solubilisation. The protein was concentrated with 50KDa cut-off filter concentrators prior to SEC purification. (TIF) [file pone.0071842.s002.tif]

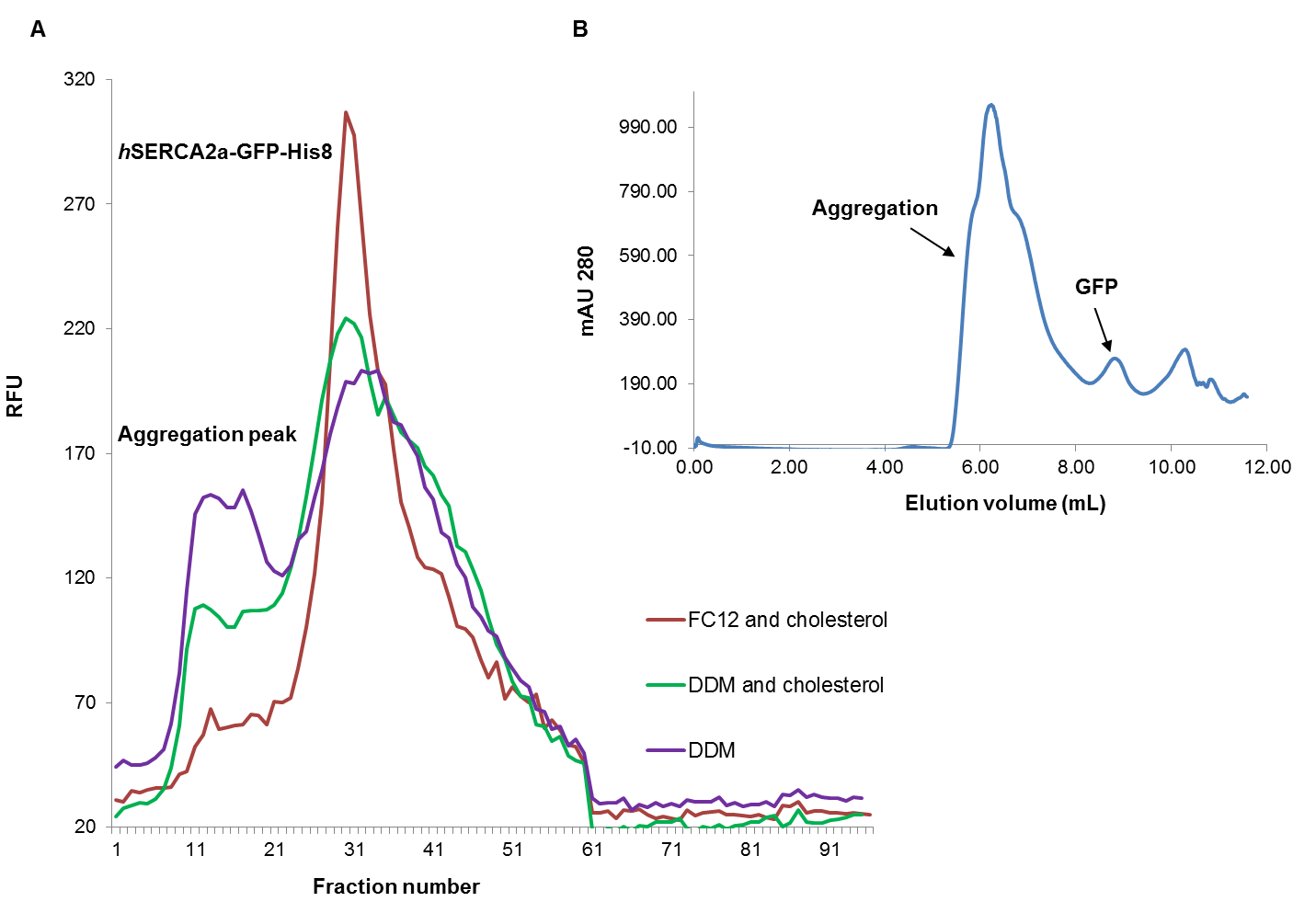

Supplement: Figure S3 — Detergent screening for hSERCA2a-GFP-His8. A. Fluorescence size exclusion chromatography profile for hSERCA2a-GFP-His8 solubilised in the presence of 2% w/v cholesterol hemisuccinate salt and 1% w/v FC12 or 1% w/v β-DDM. The same volume of solubilised hSERCA2-GFP-His8 was loaded onto a Superose 6 10/300 column for each detergent screen. The eluted fractions were analysed using a SpectraMax spectrophotometer, as described in Materials and Methods. Aggregation peak corresponds to the void volume. B. HPLC-SEC profile for large scale purification of hSERCA2a-GFP-His, in the presence of FC12 and hemisuccinate salt cholesterol. (TIF) [file pone.0071842.s003.tif]

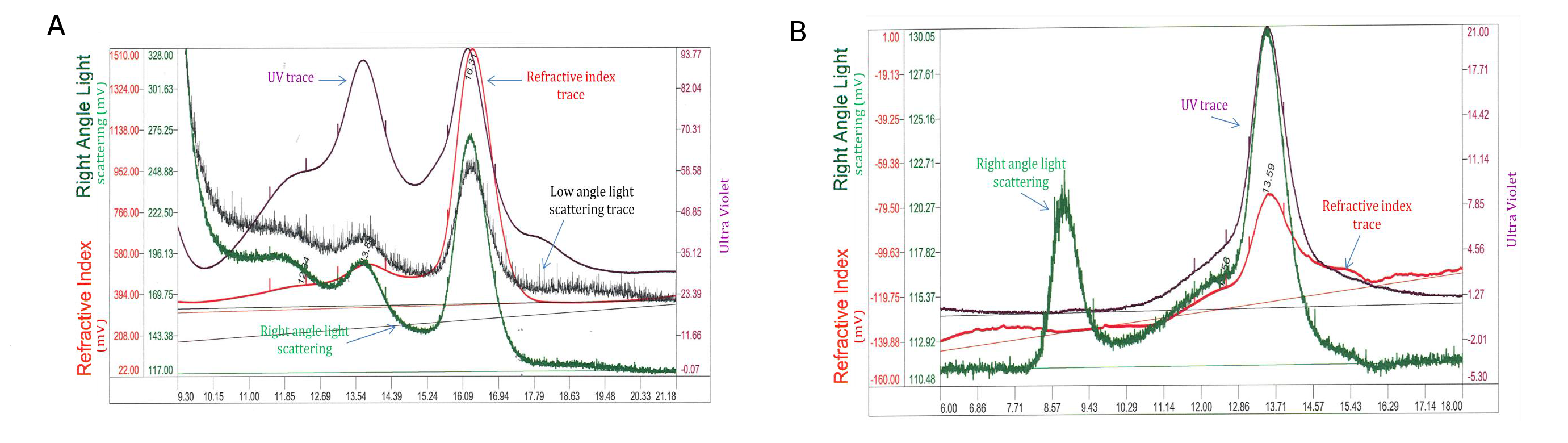

Supplement: Figure S4 — Tetra-detector analysis of purified hSERCA2a before and after SEC. The construct used for this analysis was hSERCA2a-GFP-His8. A. Protein sample analysis before SEC. The profile obtained is comparable to the SEC profiles obtained for hSERCA2a. The data shows two UV peaks after the void volume, the first peak (at 13.83mL) corresponds to hSERCA2a monomer and the second peak (at 16.31mL) presence a refractive index trace, indicating excess detergent micelles. B. Protein sample analysis after SEC. The protein fraction eluted from SEC, corresponding to the monomer form of hSERCA2a, was run on the Tetra-detector. The result indicates that the concentrated detergent micelles are separated and eliminated during SEC. The refractive index peak corresponds to the protein-detergent complex. (TIF) [file pone.0071842.s004.tif]
